# Comparison of machine learning models for the prediction of mortality of patients with unplanned extubation in intensive care units

**DOI:** 10.1038/s41598-018-35582-2

**Published:** 2018-11-20

**Authors:** Meng Hsuen Hsieh, Meng Ju Hsieh, Chin-Ming Chen, Chia-Chang Hsieh, Chien-Ming Chao, Chih-Cheng Lai

**Affiliations:** 1 Department of Electrical Engineering and Computer Science, University of California, Berkeley, Berkeley, California USA; 2 Department of Medicine, Poznan University of Medical Science, Poznan, Poland; 3 Department of Recreation and Health Care Management, Chia Nan University of Pharmacy and Science, Tainan, Taiwan; 4 Department of Intensive Care Medicine, Chi Mei Medical Center, Tainan, Taiwan; 5 Department of Pediatrics, China Medical University Children’s Hospital, China Medical University, Taichung, Taiwan; 6 Department of Intensive Care Medicine, Chi Mei Medical Center, Liouying, Tainan Taiwan

## Abstract

Unplanned extubation (UE) can be associated with fatal outcome; however, an accurate model for predicting the mortality of UE patients in intensive care units (ICU) is lacking. Therefore, we aim to compare the performances of various machine learning models and conventional parameters to predict the mortality of UE patients in the ICU. A total of 341 patients with UE in ICUs of Chi-Mei Medical Center between December 2008 and July 2017 were enrolled and their demographic features, clinical manifestations, and outcomes were collected for analysis. Four machine learning models including artificial neural networks, logistic regression models, random forest models, and support vector machines were constructed and their predictive performances were compared with each other and conventional parameters. Of the 341 UE patients included in the study, the ICU mortality rate is 17.6%. The random forest model is determined to be the most suitable model for this dataset with F_1_ 0.860, precision 0.882, and recall 0.850 in the test set, and an area under receiver operating characteristic (ROC) curve of 0.910 (SE: 0.022, 95% CI: 0.867–0.954). The area under ROC curves of the random forest model was significantly greater than that of Acute Physiology and Chronic Health Evaluation (APACHE) II (0.779, 95% CI: 0.716–0.841), Therapeutic Intervention Scoring System (TISS) (0.645, 95% CI: 0.564–0.726), and Glasgow Coma scales (0.577, 95%: CI 0.497–0.657). The results revealed that the random forest model was the best model to predict the mortality of UE patients in ICUs.

## Introduction

Acute respiratory failure is a common clinical condition in the intensive care unit (ICU), and most patients with acute respiratory failure require endotracheal intubation with mechanical ventilation (MV) support. For these patients, both endotracheal tube and mechanical ventilator are essential for life support devices. However, unplanned extubation (UE) – an accidental removal of an endotracheal tube (ETT), may develop in about 2–16% of acute respiratory failure patients requiring MV^1–8^. Although some UE patients may have successful extubation without re-intubation, other patients experiencing UE may have severe complications, such as aspiration pneumonia, unstable hemodynamic status, airway obstruction, bronchospasm, respiratory failure, prolonged MV uses, and a prolonged length of stay in ICUs and hospitals^9–13^. Most unfortunately, some UE patients may present a fatal outcome; the mortality rate can even be as high as 25%^7,13,14^.

Several studies reported that tachypnea before UE, underlying uremia or liver cirrhosis, severe conditions with higher APACHE II scores, not undergoing the weaning process, reintubation, chronic neurological disease, and emergency surgery were found to be significantly associated with high mortality among UE patients^4,15^. Since clinical assessment of illness severity is especially important for the prediction of the mortality and morbidity of critically ill patients, several researchers have attempted to design various scoring systems to determine illness severity for the prediction of patient prognosis. The Acute Physiology, Age, Chronic Health Evaluation (APACHE-II) score predicts the mortality risk for critically ill patients in the ICU^16^, whereas the Therapeutic Intervention Scoring System (TISS) assesses illness severity. Both systems are often used in conjunction to assess patient prognosis^17^. The usage of these scales, however, comes with limitations. The TISS-28 includes 28 items, divided into seven groups: basic activities, ventilatory support, cardiovascular support, renal support, neurological support, metabolic support, and specific interventions^18^. However, its accuracy in predicting actual mortality, alongside the APACHE II system, is limited^19^.

In recent years, multivariate outcome prediction models such as artificial neural networks (ANN), logistic regression models (LR), random forest models (RF), and support vector machines (SVM) have been developed in many areas of health care research^20–25^. In this study, we aim to construct several machine learning models to predict the mortality of UE patients and compare their predicting performance with other conventional parameters.

## Materials and Methods

### Patients and setting

This study was conducted in eight adult ICUs of Chi-Mei Medical Center from December 1, 2008 through July 31, 2017. This is a 1288-bed tertiary medical center with 96 ICU beds: 48 medical ICU beds, 9 cardiac beds, and 39 surgical beds for adults. Every year, an average of more than 5,000 patients are admitted to the ICU. The ICU is covered by intensivists, senior residents, nurses, respiratory therapists, dietitians, physical therapists, and clinical pharmacists. Each shift had the same workload and the patient-to-nursing staff ratio is of 2:1. There were no differences in nursing experience by shift. Each respiratory therapist was responsible for fewer than 10 patients at the same time on every shift. The ICU team made rounds at least once daily, and respiratory therapists were responsible for all the weaning processes and spontaneous breathing trials of all MV patients. An UE was defined as the dislodgement or removal of the ETT from the trachea in a patient undergoing invasive MV at a time that was not specifically planned for or ordered by the physicians in charge of the patient. During the study period, a total of 341 patients experiencing UE were enrolled in this study, and their demographic and clinical information, laboratory results, comorbidities, severity scores, mortality, and length of stays for both ICU and hospital were collected for analysis. Our elderly patients (≥65 years) were about 58.7% (201/341) of all UE patients. The data were retrospectively collected and then analyzed. Therefore, informed consent was specifically waived and the study was approved by the Institutional Review Board of Chi Mei Medical Center (IRB: 10705–011). All methods were performed in accordance with the relevant guidelines and regulations.

### Constructing data sets

All features are extracted from the original dataset. The categorical data is cleaned and one-hot encoded in RStudio. The age data, which is continuous, is unity-based normalized and standardized. After data processing, there are 16 input features. The features include subject sex, age, APACHE II scores, Glasgow Coma Scale (GCS), TISS scale, comorbidities, ICU length of stay in days, and hospital length of stay in days. These features are chosen due to their wide availability in ICUs.

### Data description

The entire data set is comprised of 341 data points. The data is split into training and test sets at an approximate 9:1 ratio. 307 subjects are placed into the train set while 34 subjects are placed in the test set. In the overall dataset, the data distribution is unbalanced: ratio between patient death and survival is 1:4.683. To ensure that the output of the prediction model does not overfit the data, the data is weighted according to their outcome ratios when training the models.

### Algorithm and training

The configuration of each model was reached through a hyperparameter selection process using the *k*-fold cross-validation accuracy (*k* = 10). We decided to use *k*-fold cross-validation instead of holdout cross-validation due to the limited number of subjects. The hyperparameter selection process was independently performed for each model type.

#### Artificial Neural Network Model

We began the model construction process by testing a three-layer perceptron network with a hidden layer that has half the number of neurons compared to the input layer. Using this configuration, we tested various activation functions in the hidden layer (Rectified Linear Unit (ReLU), Scaled Exponential Linear Unit (SeLU), and inverse tangent (*tanh*)) and the output layer (Softmax and sigmoid). If the model was under-fitting, we increased the number of hidden layers and neurons. If the model was over-fitting, we added regularization or decreased the number of hidden layers and neurons. Once the model achieved high precision and recall, we applied various optimizers (Root Mean Square Propagation (RMSProp), Adam, Adadelta, Adam with Nesterov Momentum, and PowerSign) to the same architecture and determined if the model would perform better. We would decrease the learning rate if the model did not converge, but this was not found necessary.

The final ANN model consists of one input layer of 16 dimensions, a hidden layer of 24 dimensions, and an output layer of 2 dimensions. The network is trained using stochastic gradient descent and optimized using Adam with default parameters outlined by Kingma *et al*.^26^. The neural network is trained for 200 epochs. We used the SeLU activation function at each layer and the Softmax at the output layer^27^. Dropout regularization of 20% is applied at the input layer and 50% at the output layer^28^. The categorical cross entropy error function for binary classification is used as the loss function. The ANN model is implemented using the Tensorflow framework (version 1.9.0)^29^.

#### Logistic Regression Model

First, we used the default configuration to determine the best optimizer. We tested different solvers, which include the Newton-conjugate gradient method (Newton-CG), Limited-memory Broyden–Fletcher–Goldfarb–Shanno (L-BFGS) algorithm, stochastic average gradient descent, and the liblinear solver, and compared their performances. Then, we trained models with different regularization strengths on a linear scale.

The final LR model used L_2_ regularization with primal formulation. Primal formulation was used because there are more samples than features. Stochastic average gradient descent was used as the optimizer. The one-vs-rest scheme was used as the loss function. The regularization strength was set to 1.0, and the model was trained for 100 iterations before convergence. The LR software was implemented using the scikit-learn library (version 0.19.1)^30^ and the LIBLINEAR library (version 3.21)^31^.

#### Random Forest Model

First, we trained the data on a single decision tree model to determine the optimal depth. We started with a depth of one and increased the depth until the model began to overfit, or when the precision and recall of the train and test sets began to diverge. Then, we trained random forest models with various number of trees. We started with one tree and increased the number of trees until the out-of-bag error did not decrease further.

The final RF model used ten separate decision tree estimators. Each decision tree used Gini impurity to measure the quality of split. The minimum number of samples required to split a node was set to two, and the minimum samples per leaf is set to one. All trees had a maximum depth of four; this was done to prevent the model from overfitting the training set. Probability estimates were used to plot the ROC curve. The RF model was implemented with the scikit-learn framework (version 0.19.2)^30^.

#### Support Vector Machine Model

The SVM model is a C-support vector classification (C-SVC) model. We began by testing out various kernel types (linear, polynomial, sigmoid, and radial-basis function kernels) using the default kernel coefficient (gamma) and C value. We tested the polynomial kernel with degree three. Then, we trained different models with varying gamma and C values on a logarithmic scale.

The final SVM model used a radial basis function (RBF) as its kernel with the shrinking heuristic enabled. The model used a C value of one and a gamma value of the reciprocal of the number of features. Additionally, probability estimates were calculated in order to plot a ROC curve for the model. The SVM model was implemented using the LIBSVM library (version 3.21)^32^.

### Statistical analyses

Mean values, standard deviations, and group sizes were used to summarize the results for continuous variables. The differences between the survival and non-survival group at hospital discharge were examined by univariate analysis with a Student *t* test and a Chi-square test. A *p* value < 0.05 was considered statistically significant. Statistical analysis of the data was done with SPSS 13.0 for Windows (SPSS, Inc., Il, USA).

Since the data distribution is unbalanced, accuracy is not a reliable measurement of prediction model performance^33^. Instead, we used the weighted averaged F_1_, precision and recall values to measure model performance. These three metrics are calculated for the train set, the test set, and all data.

The Receiving Operating Characteristic (ROC) curve is also used as a metric to measure prediction model performance. The area under ROC curve (AUROC) of each prediction model was pairwise-compared using the DeLong test^34^. The area under ROC curve of the prediction models were also compared to the those of the control predictors of the original data set, which include the APACHE II score, GCS, and TISS scale.

## Results

### Clinical features of UE patients

Table 1 shows the demographic and clinical characteristics of the study population. Of the 341 patients included in the study, 67.1% were male and 32.8% were female. The mean age of survivors was 64.96 years, while the mean age of non-survivors was 66.85 years. The ICU mortality rate is 17.6%. The mean APACHE II score among non-survivors is 24.23, which is significantly higher than that of the survivors, at 15.92 (*p* < 0.001). The non-survivor group had higher risks of underlying cancer, liver cirrhosis and uremia than the survivor group. The mean length of stay in ICU and hospital were 13.7 days and 36.12 days respectively for the survivor group, and 21.66 and 44.55 days respectively for the non-survivor group.

**Table 1:** The demographic and clinical characteristics of ICU patients.

Variable	Survivor n = 281	Non-survivor n = 60	*P value*
Sex (male/female)	183/98	46/14	
Age, year	64.96 ± 16.86	66.85 ± 14.36	0.420
BMI	23.56 ± 4.33	23.47 ± 5.02	0.894
APACHE II scores	15.92 ± 7.83	24.23 ± 8.55	<0.001
Glasgow Coma scales	10.46 ± 3.73	9.50 ± 4.13	0.076
TISS scales	26.75 ± 8.00	31.00 ± 9.21	0.001
Comorbidities			
Cancer	18 (6)	12 (20)	0.001
COPD	41 (15)	7 (12)	0.554
Coronary artery disease	69 (25)	14 (23)	0.841
Liver cirrhosis	5 (2)	10 (17)	<0.001
Uremia	14 (5)	13 (22)	<0.001
Stroke	92 (33)	13 (22)	0.092
Diabetes	67 (24)	20 (33)	0.126
ICU days	13.70 ± 11.64	21.66 ± 20.79	<0.001
Hospital days	36.12 ± 26.64	44.55 ± 41.99	0.049

Data are presented as mean ± standard deviation or n (%).

BMI = Body Mass Index (kg/m^2^).

APACHE = Acute Physiology and Chronic Health Evaluation.

TISS = Therapeutic Intervention Scoring System.

COPD = Chronic obstructive lung disease.

ICU = intensive care unit.

### Results of Prediction Models and Control Predictors

The F_1_, precision, and recall values of all models are shown in Table 2. In the test set, the RF model has the greatest F_1_ value among all models, followed by the SVM, ANN, and LR models. The RF model also has the greatest recall and precision values among all models in the test set.

**Table 2:** F_1_, Precision, and Recall values for all prediction models.

Dataset	Metric	ANN	LR	RF	SVM
All	F_1_	0.819	0.819	0.863	0.831
	Precision	0.846	0.848	0.888	0.848
	Recall	0.805	0.805	0.853	0.821
Train	F_1_	0.829	0.706	0.824	0.775
	Precision	0.840	0.788	0.824	0.790
	Recall	0.824	0.676	0.824	0.765
Test	F_1_	0.820	0.808	0.860	0.825
	Precision	0.844	0.842	0.882	0.842
	Recall	0.806	0.792	0.850	0.815

**Figure 1:**
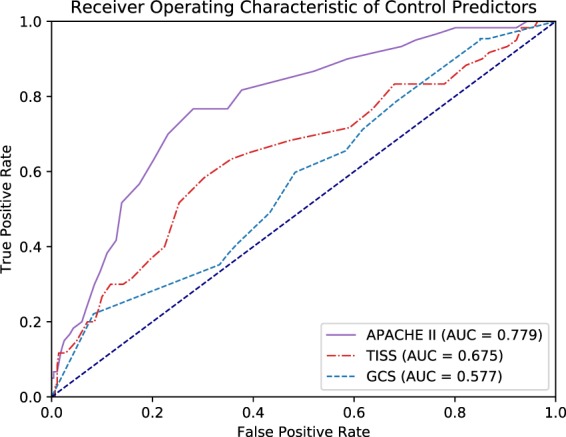
ROC curve of control variables.

**Figure 2:**
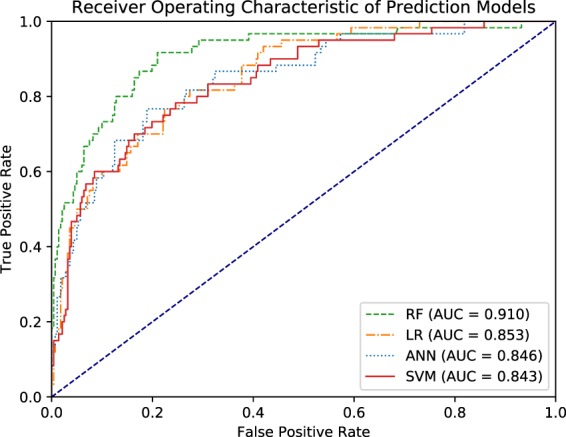
ROC curve of ANN, LR, RF, and SVM models.

The area under ROC curves of the control predictors are outlined in Table 3 and Fig. 1. The area under ROC curves of all control predictors are significantly greater than the null hypothesis area of 0.5. The area under ROC curve for all prediction models is summarized in Table 4 and Fig. 2. The RF model had the highest area under ROC curve among all prediction models. There was no significant difference between any of the prediction models using the standard 95% confidence interval criteria. However, the *p* values between the RF model and all other models (*p* ≈ 0.08) were lower than the other comparisons.

**Table 3:** Area under ROC of Control Variables.

Control Variable	AUROC	SE	95% CI
APACHE II scores	0.779	0.032	0.716–0.841
Glasgow Coma Scales	0.577	0.041	0.497–0.657
TISS scales	0.645	0.041	0.564–0.726

AUROC = Area under ROC curve.

SE = Standard Error of area under ROC curve.

CI = Confidence interval.

**Table 4:** Area under ROC curve for all prediction models.

Model	AUROC	SE	95% CI
ANN	0.846	0.028	0.791–0.902
LR	0.853	0.025	0.804–0.903
RF	0.910	0.022	0.867–0.954
SVM	0.843	0.028	0.787–0.898

AUROC = Area under ROC curve.

SE = Standard Error of area under ROC curve.

CI = Confidence interval.

The area under ROC curves were also pair-wise compared with those of the control variables. The RF model was the only model that had a significantly better prediction power than APACHE II, GCS, and TISS according to the area under ROC curve (*p* < 0.0001) (Table 5). The ANN, LR, and SVM models have a significantly better prediction power than GCS and TISS and does not have a significantly better prediction power than APACHE II (Table 6).

**Table 5:** Pairwise *p* values of area under ROC curves of prediction models using the DeLong test.

AUROC	ANN	LR	RF	SVM
ANN	1	0.844	0.085	0.912
LR	0.844	1	0.086	0.792
RF	0.085	0.086	1	0.074
SVM	0.912	0.792	0.074	1

**Table 6:** Pairwise *p* values of area under ROC curves of prediction models and the control variables using the DeLong test.

Control Variable	ANN	LR	RF	SVM
APACHEII	0.085	0.063	0.002	0.101
GCS	<0.0001	<0.0001	<0.0001	<0.0001
TISS	<0.0001	<0.0001	<0.0001	<0.0001

## Discussion

In terms of the F_1_ value across all data, the RF model achieves the best performance among all models. The RF model is also the only model that performed significantly better than APACHE II, GCS, and TISS according to the area under ROC curve. The RF model has F_1_ 0.860, precision 0.882, and recall 0.850 in the test set, and an area under ROC curve of 0.910 (SE: 0.022, 95% CI: 0.867–0.954). Therefore, in this study, we demonstrated that a random forest model is a good predictor of UE patient mortality.

We showed that when a model with a combination of multiple existing physiological scores, including APACHE II, GCS, TISS scores and eight comorbidities, are analyzed using a random forest model, the outcome of death or survival can be predicted reasonably. The usage of random forests in this study opens the feasibility for an aggregate index for a reliable patient prognosis modelling using existing scoring systems. Furthermore, the model used in this study includes features that are not included in standard APACHE II scoring such as chronic liver disease, which in Table 1 shows a significant difference between survivors and non-survivors. Ultimately, the incorporation of multiple ICU scoring systems in our proposed model shows its flexibility to extend and enhance existing frameworks for better prognosis prediction.

Several studies had tried to compare the predictive power in ICU mortality between different machine learning models^35–37^. In a medical-neurological Indian ICU, Nimgaonkar *et al*.^35^ showed that an ANN using 15 variables was superior to APACHE II in predicting hospital mortality (p < 0.001). Another study using the University of Kentucky Hospital’s data showed that the performance of ANN was as good as APACHE II in predicting ICU mortality^36^. Though this study had similar findings in that this ANN model had a slight edge over APACHE II, TISS, and GCS, for predicting ICU mortality, this study further compared among different machine learning models including ANN, LR, RF, and SVM models and found that the RF model results in the highest AUROC, hence the best predictive power. It indicates that RF model may be a better machine learning method for prediction of the outcome of UE patients. In summary, while all of the tentative models can help in predicting the outcome of ICU patients, even for a specific group – UE patients; the RF model may even outperform conventional predicting tools, such as APACHE scores.

In this study, the overall mortality rate of UE patients was 17.6%, and the mortality cases had more underlying cancer, liver cirrhosis, and uremia. All these findings are consistent with the previous study^4^, and remind intensivists that they should be aware of the high mortality of UE patients, especially for the patients with multiple co-morbidities. In contrast to mortality cases, more than 80% of patients had successful extubation and survival-to-discharge. This finding might hint that the extubation was delayed in these cases with successful UE. The possible cause of delayed extubation may be due to that the final decision regarding extubation should be made by intensivists even though hospitals have standard weaning and extubation protocols.

## Conclusion

The results revealed that the random forest model was the best model for predicting the mortality of UE patients in the ICUs. Such a model will be helpful for predicting ICU patients’ mortality.

Limitations of our study include the lack of more patient data and features. Goodfellow *et al*. recommends that a supervised deep learning algorithm will generally achieve acceptable performance with more than 5000 data points^38^. However, this study shows that we can still develop a good prediction model using a limited data set. Future studies can be performed to determine whether similar datasets with a larger number of samples will produce comparable results. In addition, clinical lab data, such as liver and renal function, are omitted because these features fluctuate frequently during the patient’s stay. We chose features that were consistent for each patient and were known to predict patient mortality, which could yield good results according to this study.
